# Interlaminar Mechanical Properties and Toughening Mechanism of Highly Thermally Stable Composite Modified by Polyacrylonitrile Nanofiber Films

**DOI:** 10.3390/polym14071348

**Published:** 2022-03-26

**Authors:** Yingjian Ma, Yangpeng Zhuang, Chunwei Li, Chuyang Luo, Xing Shen

**Affiliations:** 1 State Key Laboratory of Mechanics and Control of Mechanical Structures, Nanjing University of Aeronautics and Astronautics, Nanjing 210016, China; summeryuyi2008@163.com; 2 Shanghai High Performance Fibers and Composites Center (Province-Ministry Joint), Center for Civil Aviation Composites, Donghua University, Shanghai 201620, China; 2200443@mail.dhu.edu.cn; 3 AVIC General Huanan Aircraft Industry Co., Ltd., Zhuhai 519042, China; 18688170899@163.com

**Keywords:** composites, electrospinning, interlaminar strength, nanofiber film, toughening mechanism

## Abstract

This work concentrated on the interlaminar mechanical properties and toughening mechanism of carbon fiber-reinforced bismaleimide resin (CF/BMI) composites modified by polyacrylonitrile (PAN) nanofiber films. The PAN nanofiber films were prepared by electrospinning. End-notched flexure (ENF) and short-beam strength tests were conducted to assess the mode II fracture toughness (G_IIc_) and interlaminar shear strength (ILSS). The results showed that the G_IIc_ and ILSS of PAN-modified specimens are 1900.4 J/m^2^ and 93.1 MPa, which was 21.4% and 5.4% higher than that of the virgin specimens (1565.5 J/m^2^ and 88.3 MPa), respectively. The scanning electron microscopy (SEM) images of the fracture surface revealed that the PAN nanofiber films toughen the composite on two scales. On the mesoscopic scale, the composite laminates modified by PAN formed a resin-rich layer with high strength and toughness, which made the crack propagate across the layers. At the microscopic scale, the crack propagation between two-dimensional nanofiber films led to constant pull-out and breakage of the nanofibers. As a result, the interlaminar fracture toughness of the composite laminates improved.

## 1. Introduction

Carbon fiber (CF)-based composites have been widely used in the aerospace field because of their outstanding advantages, such as high specific strength, high specific stiffness, excellent fatigue strength, and environmental stability [1,2]. However, their interlaminar shear strength (ILSS) and fracture toughness were insufficient, which hinder their application in aircraft for weight reduction. Therefore, it is necessary to improve the ILSS and fracture toughness properties of CF-based composites. According to the literature review, there are three methods to improve the toughness of CF-based composites, including matrix toughening (chemical [3] and physical [4,5] modification methods), Z-direction toughening (Z-pin [6], stitching [7,8,9], 2.5D or 3D weaving [10]), and interlaminate toughening (particle toughening [11], fiber toughening [12], and film toughening [13]). Matrix toughening could improve the in-plane and interlaminar toughness of composites simultaneously, but it also brings changes to the viscosity, glass transition temperature (T_g_), and thermal properties of the resin, which would affect the manufacturing process of CF-based composites [14]. Z-direction toughening, such as Z-pin and stitching, forms bridging structures in the interlaminar region of the composites to achieve an obvious toughening effect, but the in-plane properties would reduce to a certain extent [15]. In particular, the in-plane performance of composites using the 3D weaving method decreases significantly compared with that of typical laminates [16]. Although such a method provides an obvious toughening effect, the long experimental process cycle, complex operation procedures, and relatively high manufacturing cost limit their usage in practical applications.

Interlaminar toughening refers to the method that inserts a special discrete layer material into the interlaminar region of the composites. This special discrete layer could hinder the generation of cracks and prohibit crack propagation. By applying this method, the interlaminar fracture toughness of the composites can be greatly improved without significantly adjusting the original manufacturing process. Nanofiber films are widely used in interlaminar toughening of composites because of their excellent properties, such as high specific surface area, high porosity, and thin thickness. In recent years, thermoplastic nanofiber films prepared by electrospinning technology have attracted extensive attention for the improvement of interlaminar toughness [17,18,19]. Different properties of nanofiber films can be obtained by adjusting the electrospinning parameters, such as spinning solution, spinning speed, spinning distance, and voltage, etc.

Zheng et al. [20] found that PA66/PCL composite nanofiber films increased the mode I and mode II interlaminar fracture toughness of CF/epoxy composites by 98% and 101%, respectively. Subagia et al. [21] found that the flexural strength and modulus of the composites were significantly improved by inserting polyurethane nanofiber films. Cai et al. [22] prepared blended polysulfone (PSF)/cellulose nanocrystal (CNC) nanofiber films by electrospinning and then inserted them into CF/epoxy composites. The results showed that the mode I and mode II interlaminar fracture toughness were improved. Saeedifar et al. [23] studied the toughening effect of electrospun PA66 nanofiber films on CF/epoxy composite laminates under mode II high-temperature loading. It was found that the mode II interlaminar fracture toughness (G_IIC_) of nanofiber film-modified composites was quadrupled over the unmodified counterparts at room temperature. However, although G_IIC_ did not change from room temperature to 100 °C, it decreased by 34% and 43%, respectively, by further heating to 125 °C and 160 °C. Taheri et al. [24] prepared electrospun nanofiber films containing polyvinyl butyral (PVB) and pyrolytic carbon (PyC), which were interleaved between the layers of the laminates. The toughening behavior of the modified laminates was studied by a mode II fracture test. The results showed that the incorporation of the pure PVB nanofiber films was insufficient in greatly improving the fracture toughness of laminates (~7%). In contrast, the fracture toughness was improved by ~24% by adding (4.76 wt%) PyC particles into the PVB nanofiber films. Polyacrylonitrile (PAN) is a low-cost homopolymer with good toughness and ductility. The strong polarity of cyano (-CN) groups endows it with good adhesion and interfacial compatibility with resin and provides excellent weather, light, and solvent resistance [25,26,27]. Electrospun PAN nanofiber films are ideal materials for interlaminar toughening due to their excellent strength, modulus, and toughness [28,29]. Although many studies have been carried out on the interlaminar toughening of electrospun nanofiber films, the current research mainly focused on PA66, PCL, PSF, PVB, etc., [17,18,19,20,21,22,23,24,30]. There are few reports on the interlaminar mechanical properties of PAN nanofiber film-toughened composites. Herein, PAN nanofiber films were prepared by electrospinning, and the PAN nanofiber films were incorporated into CF prepregs. The CF/bismaleimide resin (CF/BMI) composites were fabricated by a vacuum bagging process. The mode II fracture toughness and ILSS of the composites before and after modification were tested, and the toughening mechanism was also analyzed accordingly.

## 2. Experiments

### 2.1. Materials

Polyacrylonitrile (PAN, M_W_ = 51,000) and N, N-dimethylformamide (DMF) solutions were supplied by RHAWN Chemical Technology Co., Ltd., Shanghai, China. The CCF800H/AC631 unidirectional prepreg was provided by AVIC Composite Co., Ltd., which was composed of CF (CCF800H, Weihai Tuozhan Fiber Co., Ltd., Weihai, China) and bismaleimide resin (AC631, AVIC Composite Co., Ltd., Beijing, China). The properties of CF, BMI resin, and prepreg are given in Table 1. The average tensile modulus and strength of CCF800H CF are 293 GPa and 5641 MPa, respectively. The glass transition temperature and decomposition temperature of AC631 resin are 240 °C and 466 °C, respectively. The densities of CCF800H and AC631 are 1.78 g/cm^3^ and 1.2 g/cm^3^, respectively. The area density of the CF is 133 ± 2 g/m^2^, and the resin content of the prepreg is 33 ± 5 wt%. Additionally, the nominal ply thickness of the prepreg is 0.125 mm.

### 2.2. Preparation of PAN Nanofiber Films

**Figure 1:**
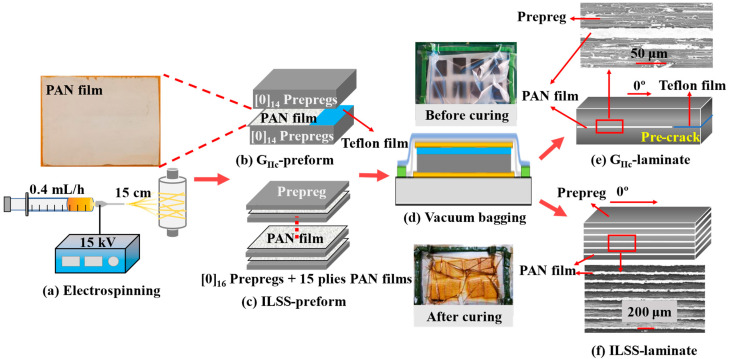
Illustration of the preparation process of the composites containing PAN nanofiber films (**a**–**f**).

PAN was dissolved in DMF to prepare a mixed solution with a mass fraction of 10%. The solution was stirred on a magnetic stirrer at a speed of 1000 rpm for 8 h at 55 °C to obtain a yellow transparent precursor. Afterward, the PAN nanofibers were electrospun from the precursor using an in-house developed single-nozzle electrospinning machine. The electrospinning process (e.g., the concentration of spinning solution, spinning voltage, feed rate, tip-to-collector distance, speed of rotating collection, etc.,) has a great effect on the properties of nanofiber films. On the one hand, when the concentration of polymer solution is too low, the degree of molecular chain entanglement is weak, and thus, it is easy to form beads on the fiber surface. In contrast, when the concentration is too high, the diameter of the spun nanofibers is too large, resulting in a decrease in the specific surface area, thereby weakening its binding with the matrix [31,32]. On the other hand, when the spinning voltage is too low, the polymer solution cannot form jets. When the voltage is too high, the charge density on the surface of the jet is too large, which easily causes current disorder and jet instability [33]. Moreover, only within a certain range of feed rates can the stability of the Taylor cone be ensured, as well as the solvent evaporation, molecular chain stretching, and crystallization of the polymer solution jet [34,35]. Finally, with an appropriate spinning distance and spinning voltage, the fiber diameter can be reduced, and the fiber can be fully stretched in the electric field to improve the crystallinity of nanofibers, which is beneficial to enhancing the mechanical properties of nanofibers [36]. Therefore, the optimized spinning process used in this work was as follows: the feed rate was 0.4 mL/h, and the voltage was 15 kV. The tip-to-collector distance was 15 cm, and the speed of rotating collection was 400 rpm (see Figure 1a). First, the PAN solution was electrospun directly onto silicone release paper for 6 h in a conditioned room at 20 ± 2 °C and 40 ± 5% RH. Second, the PAN nanofiber film was removed from the silicone release paper. Finally, the PAN nanofiber film was fully dried in an oven at 60 °C for 12 h.

### 2.3. Preparation of CF/BMI Composites

The preparation process of the laminates interleaved by PAN nanofiber films is shown in Figure 1. Two types of laminates, namely, G_IIC_-laminate and ILSS-laminate, were prepared by a vacuum bagging process. For the G_IIC_-laminate, a 15 μm Teflon film (to create the initial crack) and a PAN nanofiber film (named F) were interleaved between the 14th and 15th layers synchronously (see Figure 1b). For the ILSS-laminate, the PAN nanofiber films were cross-stacked between two layers of prepreg (named P) (see Figure 1c). Therefore, the G_IIC_-laminate was composed of 28-ply CCF800H/AC631 prepregs and 1-ply PAN nanofiber film (i.e., [P]_14_ + [F] + [P]_14_) with a total thickness of 3.5 mm. The ILSS-laminate was composed of 16 ply CCF800H/AC631 prepregs and 15 ply PAN nanofiber films (i.e., the layup sequence is [P/F/P/F/P/F/P/F/P/F/P/F/P/F/P/F/P/F/P/F/P/F/P/F/P/F/P/F/P/F/P]) with a total thickness of 2.2 mm. The interactions between the layers were realized by resin bonding. First, the CCF800H/AC631 prepregs and PAN nanofiber films were draped on a plane mold according to the layup scheme mentioned above. The PAN nanofiber films were placed on the prepreg surface, and then the silicone paper was carefully peeled off. Since the fully dried films did not adhere to the silicone paper, the PAN nanofiber films can be easily transferred to the prepreg surface. The advantage of this method compared to electrospinning prepreg directly is that there is no interaction between the solvent and the resin. Second, the G_IIC_-preform and ILSS-preform were encapsulated in a vacuum bag. Finally, the preforms were heated to 125 °C at a rate of 5 °C/min and held for 1 h with a vacuum pressure of 0.098 MPa in an oven. After that, the temperature was subsequently heated to 180 °C and 200 °C at the same rate and held for 2 h and 6 h, respectively. The mold was then cooled to room temperature, and consolidation was completed (see Figure 1d–f).

### 2.4. Mechanical Testing Procedure

**Figure 2:**
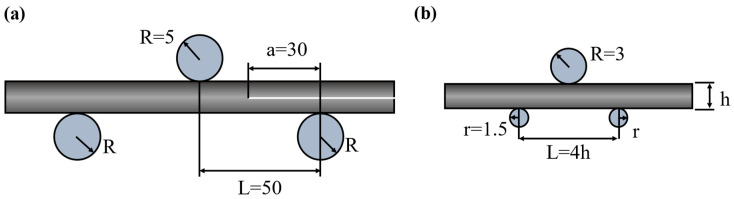
Three-point bending test configurations for (**a**) G_IIC_ and (**b**) ILSS (unit: mm).

The end-notched flexure (ENF) and short-beam strength (SBS) were tested under quasi-static loading with a constant displacement rate of 1 mm/min using a universal testing machine (ETM 105D, Wance Testing Machine Co., Ltd., Shenzhen, China) according to ASTM D7905 and ASTM D2344, respectively. All fracture tests were conducted at room temperature and in an atmospheric environment with at least five specimens. According to ASTM D7905, the specimen dimensions of G_IIC_ are 160 mm × 25 mm × 3.5 mm (length × width × thickness). The span length is 50 mm, and the effective crack length is 30 mm (see Figure 2a). The mode II fracture toughness can be calculated as follows [37]:

(1) $$\begin{array}{c} G_{\mathrm{IIC}}=\frac{9a^{2}P\delta }{2w\left(3a^{3}+2L^{3}\right)}\times 10^{3} \end{array}$$

where G_IIC_ is the mode II fracture toughness (J/m^2^). P and δ are the load (N) and deflection (mm) at the beginning of crack propagation, respectively. W is the width of the specimen (mm). a is the effective length of the crack (mm). L is the half span length (mm). According to ASTM D2344, the specimen dimensions of the ILSS are 20 mm × 6 mm × 2 mm (length × width × thickness). The span length is 8 mm (see Figure 2b). The ILSS can be calculated as follows:

(2) $$\begin{array}{c} \tau_{s}=\frac{3P_{\max}}{4\mathrm{wh}} \end{array}$$

where $\tau_{s}$ is the interlaminar shear strength (MPa). P_max_ is the peak load recorded in the test (N). w is the width of the specimen (mm). h is the thickness of the specimen (mm).

### 2.5. Characteristics

The morphologies of the PAN nanofiber films and the fractographs of the corresponding composites were observed by scanning electron microscopy (SEM, SU−4800, Hitachi Ltd., Tokyo, Japan). X-ray diffraction (XRD, Empyrean, Malvern Panalytical Ltd., Overijssel, The Netherlands) analysis was performed on the PAN nanofiber films using Cu Kα source radiation. The density of the CF/BMI composite was tested by a density meter with 0.0001 g/cm^3^ accuracy (MAY−ME104, METTLER TOLEDO Ltd., Greifensee, Switzerland) according to ASTM D 792. The void content of the CF/BMI composite was measured according to ASTM D 2734. Thermogravimetric analysis (TGA) of virgin laminate and PAN-modified laminate was conducted by a thermogravimetric analyzer (TGA−400, PerkinElmer, Waltham, MA, USA).

## 3. Results and Discussion

**Figure 3:**
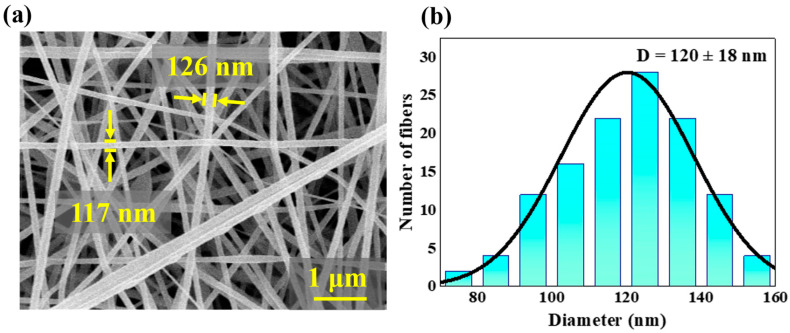
(**a**) SEM image of PAN nanofiber film; (**b**) diameter distribution of the nanofibers.

**Figure 4:**
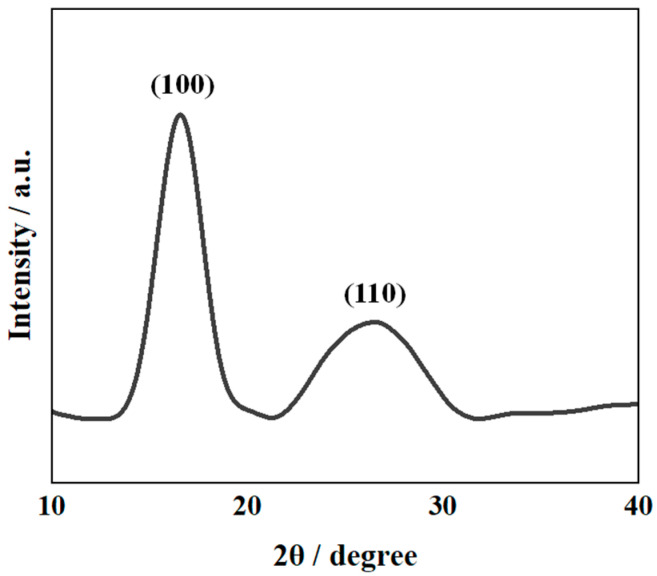
The XRD pattern of PAN nanofiber film.

Figure 3 shows representative SEM micrographs of PAN nanofiber films. The PAN nanofibers are randomly distributed with an average diameter of 120 ± 18 nm. Figure 4 shows that the XRD pattern of the PAN nanofiber film includes the (100) and (110) crystalline planes, representing the crystalline and amorphous peaks of PAN, respectively [38]. This indicates that PAN is a semicrystalline polymer with amorphous regions that can transform into crystalline regions under appropriate conditions [39].

**Figure 5:**
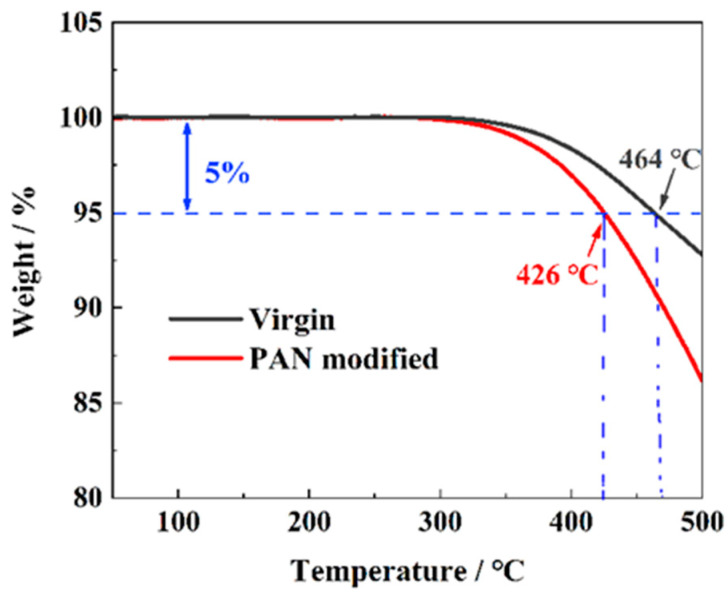
The TGA results of virgin laminate and PAN-modified laminate.

The void contents of G_IIC_-laminate and ILSS-laminate are approximately 3.12% and 4.31%, respectively. The densities of G_IIC_-laminate and ILSS-laminate are 1.511 g/cm^3^ and 1.507 g/cm^3^, respectively. The TGA results shown in Figure 5 indicate that PAN slightly reduces the heat resistance of the CF/BMI composites. This is because PAN is less heat resistant than BMI resin.

**Figure 6:**
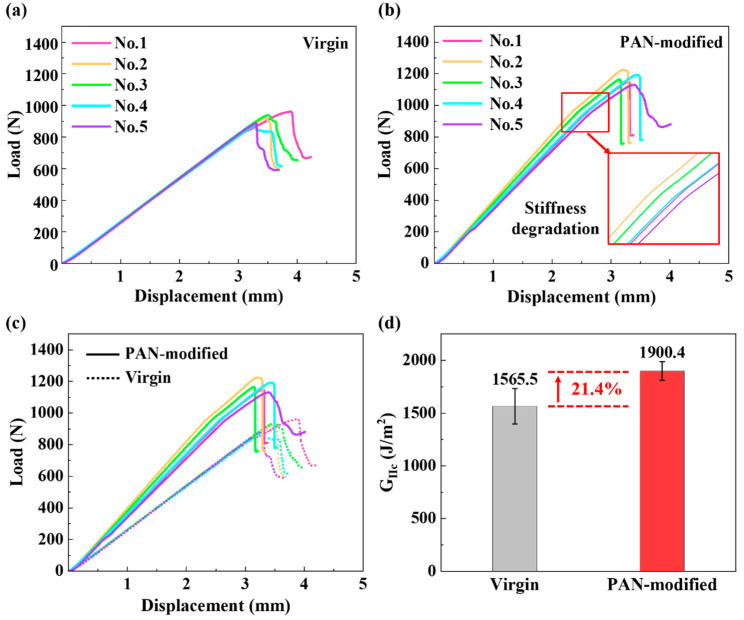
Load/displacement curves of G_IIC_ testing for (**a**) virgin laminate; (**b**) PAN-modified laminate; (**c**,**d**) comparison of G_IIC_ results.

Figure 6a–c shows the load–displacement (*P*-*δ*) curves of G_IIC_ testing for the virgin laminates and PAN-modified laminates. The results showed that the slope of the *P*-*δ* curve of PAN-modified laminates was higher, which means that the bending stiffness increased after modification by PAN nanofiber films. This can be ascribed to the fact that the modulus of the PAN nanofiber films was much larger than that of the resin. Therefore, after the PAN nanofiber film was inserted, the modulus of the original resin rich layer increased so that the bending modulus of the laminates increases. These results were consistent with previously reported results [40]. Moreover, both the maximum displacement and peak load were higher than those of unmodified laminates. The stiffness consistency of the unmodified sample is good. In contrast, the stiffness of the modified sample was slightly different with increasing load. This may be due to the uneven thickness of the electrospun film, resulting in the differences in the *P-δ* curves for the modified laminates. Additionally, for unmodified laminates, the load dropped sharply when the crack initiated. In contrast, the *P-δ* curve of the modified composite was slightly bent when the load reached approximately 1000 N, which might be caused by the deformation and fracture of interlaminar nanofibers when the crack propagated in the PAN-modified layer. The G_IIC_ value of the PAN-modified laminates increased by 21.4% (see Figure 6d). This result was consistent with the trend of other toughened materials reported in the literature [30].

**Figure 7:**
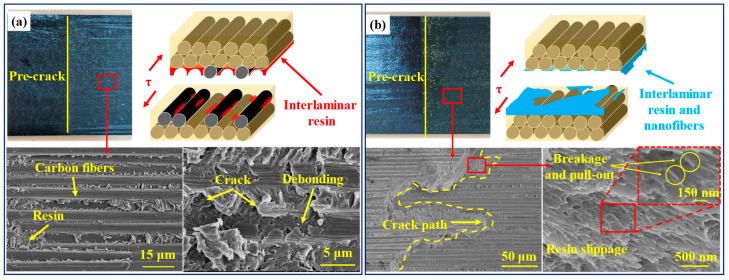
Failure morphologies of G_IIC_ for (**a**) virgin laminate and (**b**) PAN-modified laminate.

Figure 7 shows the cross-sectional facture morphologies of the two samples after the G_IIC_ test. As shown in Figure 7a, the interlaminar facture morphology of the unmodified laminates was relatively flat, and the cracks did not penetrate the interlaminar resin-rich layer. The failure mode was mainly manifested in the debonding of the fiber and the shear failure of the resin. In contrast, the morphology of the PAN-modified laminates was relatively rough (see Figure 7b). The crack propagation was observed to cross the interlaminar PAN nanofiber films up and down, which could be attributed to the strong adhesion between PAN films and resin. Moreover, it can be found at higher magnification that the resin and PAN nanofiber films were completely fused at the interlaminar crack of the modified laminates, and the diameter of PAN nanofibers decreased, forming a molten layer at the interface. When the crack propagated in the PAN nanofiber films, it had to overcome the pulling out and fracture of nanofibers as well as the shear slip of resin. The presence of the molten layer caused plastic deformation and consumed more energy; as a result, slight bending on the *P-δ* curve was observed. In conclusion, the increase in the crack propagation path and the increased energy consumption of crack propagation led to the improvement of the mode II fracture toughness of the modified composite laminates. This toughening mechanism was similar to other types of interleaving nanofiber films [17].

**Figure 8:**
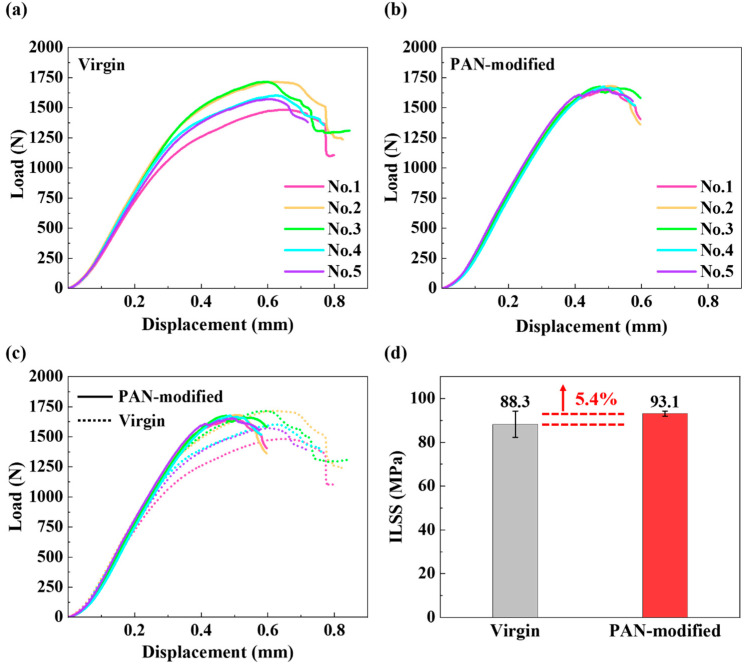
Load-displacement curves of ILSS testing for (**a**) virgin laminate; (**b**) PAN-modified laminate; (**c**,**d**) comparison of ILSS results.

Figure 8a–d shows the *P*-*δ* curves of unmodified and PAN-modified laminates in the ILSS test. It can be observed that the ILSS *P-δ* curve of unmodified laminates had high discreteness. In contrast, the ILSS *P-δ* curve of PAN-modified laminates was better. This was because the unmodified sample would have a variety of failure modes, such as interlaminar shear cracking, compression cracking, tensile failure, inelastic deformation, etc., in the short beam shear test, leading to the large nonlinearity of the *P-δ* curve before the failure of the sample. The modified samples mainly showed interlaminar shear failure. In addition, the average failure load of the PAN-modified laminates increased, but the modulus did not change. The ILSS value increased by 5.4%, an increase from 88.3 to 93.1 MPa (see Figure 8c,d), which was quite different from the observations reported in the literature [41] that PAN nanofiber films would reduce the interlaminar shear strength of the composite. Compared with the G_IIC_ test, the improvement in the ILSS value was insignificant. The reason might be that interleaved PAN nanofiber films absorbed a large amount of resin, resulting in the weakening of CF and resin.

**Figure 9:**
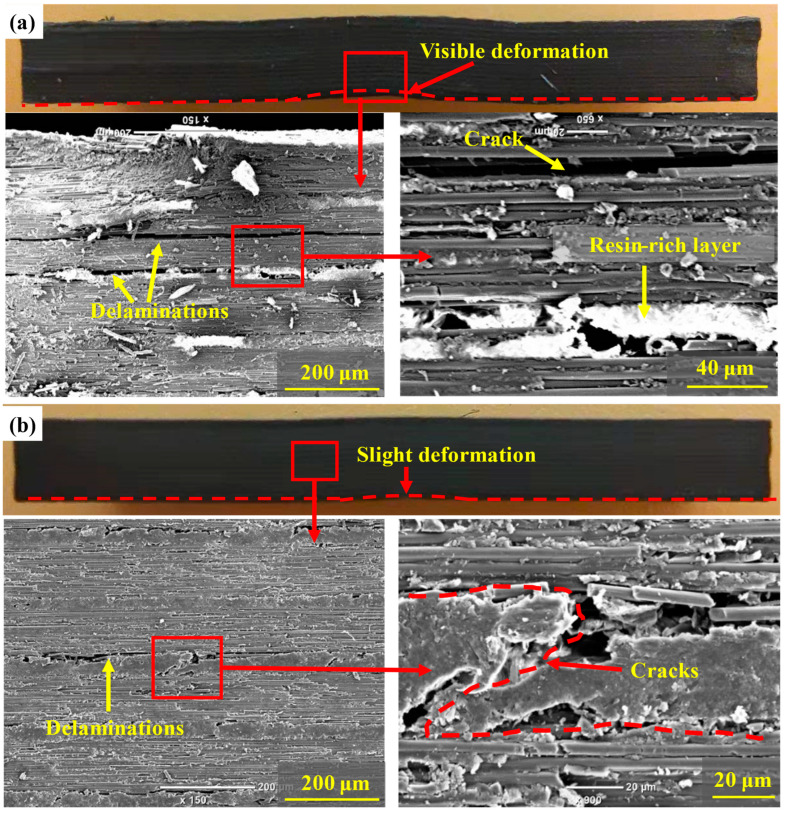
Failure morphologies of ILSS for (**a**) virgin laminate and (**b**) PAN-modified laminate.

Figure 9 shows the typical failure morphologies of the two samples after the ILSS test. The overall plastic deformation of the PAN-modified laminates weakened (see Figure 9a,b). It can be observed under low magnification that delamination occurred in the interlaminar resin-rich layer, and debonded fibers and resin in the intralaminar region were present for unmodified samples. Various failure modes were probably related to the large deformation of composite laminates. The PAN-modified laminates not only possessed higher toughness but also displayed smaller deformation. The failure mode became interlaminar cracking alone. The crack propagation alternately crossed in the interlaminar resin-rich layer, and its path ran through the whole resin-rich layer, which is beneficial to the improvement of ILSS.

**Figure 10:**
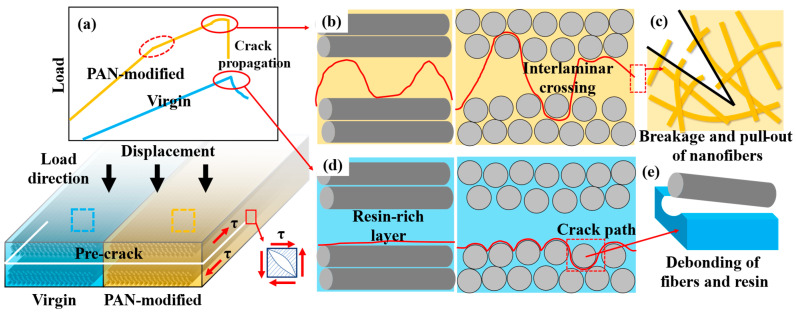
Toughening mechanism of PAN nanofiber films: (**a**) *P*-*δ* curves of virgin and PAN-modified composite; cracks propagation path for: (**b**) PAN-modified composite and (**d**) virgin composite; failure mechanism for (**b**) PAN-modified composite and (**d**) virgin composite.

According to the above analysis, the toughening mechanism of PAN nanofiber films can be summarized as follows (see Figure 10). PAN nanofiber films consist of polymer chains with -CN polar functional groups, which provide strong adhesion with resin, and their fracture toughness is better than that of unmodified resin [25,26,27]. Under the shear load, the PAN nanofiber films were subjected to shear stress in the resin-rich layer, leading to the occurrence of plastic deformation and fracture when the critical stress was exceeded (see Figure 10c). Furthermore, the PAN nanofibers become carriers of stress bridging and transfer more stress to the nearby resin owing to the two-dimensional continuous structure. The bridging effect changes the failure mode from adhesive failure to cohesive failure. This is one of the main reasons why the addition of PAN fiber films improves the interlaminar mechanical properties of the composite. Although these changes were not enough to initiate the crack in the brittle resin, a huge amount of energy was absorbed, resulting in a slight change in the slope of the *P-δ* curve (see Figure 10a red dotted circle area). When the displacement continued to increase, the stress at the crack tip increased. When the internal shear stress exceeded the bonding strength between the fiber and resin, debonding of the fiber occurred (see Figure 10e). The precrack of virgin composite laminates expanded instantaneously and propagated along the interface between the fiber and resin but did not pass through the interlaminar resin-rich layer (see Figure 10d). During the debonding of CF, a portion of the resins underwent plastic deformation due to stress concentration, causing the occurrence of cracks. As a result, the energy was absorbed gradually, leading to a slower decline of the *P-δ* curve (red circle on the blue curve in Figure 10a). The resin-rich layer in the PAN-modified samples had higher toughness. Before debonding between the carbon fiber and resin, the resin slid under the shear force and produced microcracks. The microcracks propagated along the two-dimensional nanofiber films in the interlaminar resin-rich layer, causing the pull-out and breakage of the PAN nanofibers, and thus, a large amount of energy was absorbed. This is reflected in the gradual flattening of the yellow curve in the red circle in Figure 10a. When the load reached the peak value, the microcracks instantly expanded to macrocracks and propagated along the interlaminar crossing path through the whole resin-rich layer. The failure modes mainly included the debonding of PAN films, debonding of CF, plastic deformation of resin, fracture and pull-out of PAN films. These failure modes caused the absorption of energy, resulting in a sudden drop in load. In conclusion, the composite modified by PAN nanofiber films formed a resin-rich layer with high strength and toughness. The presence of PAN nanofiber films made the cracks cross between layers. Moreover, the crack propagation between two-dimensional nanofiber films caused the constant pull-out and breakage of nanofibers. As a result, the interlaminar strength of the composite laminates improved.

In summary, the PAN nanofiber films improve the interlaminar fracture toughness by extending the crack path and increasing the strength and toughness of the resin-rich layer. This toughening method can greatly improve the interlaminar strength without reducing the in-plane performance of the composites, which is extraordinarily important to the aerospace field. In particular, the failure of the triangular zone in composite structures (such as various types of joints) is mainly due to the insufficiency of interlaminar strength [1,42]. Therefore, the application of PAN nanofiber films in the triangular zone of composite joints can effectively improve the damage resistance performance.

## 4. Conclusions

This paper focused on the toughening mechanism of PAN nanofiber film-modified CF/BMI composites. The results showed that the G_IIC_ increased by 21.4% compared with that of the virgin composite when the PAN nanofiber films were inserted. The ILSS increased by only 5.4% compared with that of the virgin composite, which might be attributed to the excessive absorption of resin by PAN films. When the PAN nanofiber film-modified composite laminates were damaged in the interlaminar region, the two-dimensional nanofiber films hindered the propagation of microcracks, causing the pull-out and breakage of nanofibers. The propagation of cracks along the interlaminar path changed the failure mode, resulting in the absorption of more energy, which in turn improved the interlaminar strength of the PAN-modified laminate.

## Figures and Tables

**Table 1:** Properties of CF, BMI resin, and prepreg.

CCF800H *		AC631 **		CCF800H/AC631 **	
Tensile modulus	293 GPa	Glass transition temperature	240 °C	Ply thickness	0.125 mm
Tensile strength	5641 MPa	5% decomposition temperature	464 °C	Resin content	33 ± 5 wt%
Density	1.78 g/cm^3^	Density	1.2 g/cm^3^	Area density of CF	133 ± 2 g/m^2^

* provided by Weihai Tuozhan Fiber Co., Ltd., Weihai, China. ** provided by AVIC Composite Co., Ltd., Beijing, China.

## Data Availability

The data used to support the findings of this study are available from the corresponding authors upon request.
